# Folic Acid as a Molecule Protecting Cells from the Negative Effects of Ultraviolet Radiation—An In Vitro Study

**DOI:** 10.3390/ph18101497

**Published:** 2025-10-05

**Authors:** Magdalena Jurzak, Paweł Ramos, Barbara Pilawa, Ilona Anna Bednarek

**Affiliations:** 1 Department of Biotechnology and Genetic Engineering, Faculty of Pharmaceutical Sciences in Sosnowiec, Medical University of Silesia, 40-055 Katowice, Poland; ibednarek@sum.edu.pl; 2 Department of Community Pharmacy, Faculty of Pharmaceutical Sciences in Sosnowiec, Medical University of Silesia, 40-055 Katowice, Poland; pawelramos@sum.edu.pl; 3 Department of Biophysics, Faculty of Pharmaceutical Sciences in Sosnowiec, Medical University of Silesia, 40-055 Katowice, Poland

**Keywords:** folic acid, ultraviolet radiation (UVR), electron paramagnetic resonance (EPR), cell cycle regulators

## Abstract

**Background:** Folic acid (FA), also known as vitamin B9, functions as a co-factor in many cellular processes. Ultraviolet radiation (UV) has been shown to cause the formation of free radicals, and chronic exposure of the skin to UV radiation has been demonstrated to result in many adverse effects. Skin protection against harmful environmental factors is one of the aims of cosmetic products. One such substance is folic acid. However, aqueous FA solutions decompose after exposure to UV radiation, and the decomposition products can exhibit variable pro/anti-oxidative roles depending on the cell type and its environment. **Objectives:** The objective of the present study was to demonstrate the effectiveness of folic acid as a UV-protective agent in vitro cell culture model. **Methods:** The experimental model comprised an in vitro culture of normal human fibroblasts derived from adult skin (NHDF-Ad). Paramagnetic electron resonance (EPR) was used to assess the interaction of folic acid with free radicals after exposure to UV radiation. RT-qPCR was utilized to evaluate the impact of ultraviolet (UV) radiation on the expression of selected cell cycle regulatory genes (*CCND1*, *P53*, *BAX*, and *BCL-2*) in vitro cultured fibroblasts that were protected by folic acid. **Results:** EPR studies revealed the antioxidant properties of folic acid. Free radical forms of folic acid are induced during UV irradiation. The strong effect of UV irradiation on interactions of folic acid with free radicals was observed. The interaction was found to be weaker for the irradiated samples. Molecular studies have demonstrated a decline in the *BAX*/*BCL-2* ratio in cells that have been treated with folic acid and exposed to UV radiation in comparison to the *BAX*/*BCL-2* ratio observed in cells that have been exposed exclusively to UV radiation and not treated with folic acid. **Conclusions:** Whilst molecular and EPR studies both confirm the effectiveness of folic acid as a UV-protective ingredient in cosmetics and pharmaceutical products, further research in this area is required.

## 1. Introduction

Folic acid*/*folate *(*pteroyl*-*L*-*glutamic acid*/*pteroyl*-*L*-*glutamate) belongs to the B group of water-soluble vitamins (B_9_) [1]. Substances that exhibit the biochemical activity of folic acid are called folacin [2,3]. Folacin consists of a pteridine base combined with one molecule each of p-aminobenzoic acid (PABA) and glutamic acid. The main active form of folate is tetrahydrofolate (H4 folate) [4,5]. Tetrahydrofolate (THF) is a carrier of active monocarbon groups: methyl, methylene, methenyl, formyl, and formimino. All of these can be converted from one form to another. THF plays a key role as a coenzyme in many metabolic reactions that occur in the human body [6]. Folic acid is essential for DNA/RNA metabolism because it plays a key role in the biosynthesis of pyrimidines and S-adenosylmethionine (SAM), which is a methyl group donor in DNA methylation [7]. SAM is also the main methyl group donor in the biosynthesis of proteins, phospholipids, hormones, and neurotransmitters [6]. A deficiency in folic acid can lead to megaloblastic anemia, multiple cancers, and cardiovascular disease. Furthermore, a lack of folate during embryogenesis can increase the risk of neural tube defects [6,8]. As folic acid affects many biological pathways, both deficiency and excess can have serious consequences. In adults, excess folic acid can cause changes in the DNA methylation profile (hypermethylation), as well as modulation of inflammation and immunity, promotion of cellular growth and proliferation, interaction with proteins and transcription factors, and changes in gene expression [9].

Data from a couple of cosmetic manufacturers shows that folic acid has regenerative properties and protects the skin against premature aging and the negative effects of ultraviolet (UV) radiation [10,11]. UV radiation is one of the environmental factors that induce changes in skin morphology and biology [12]. UV radiation induces the generation of free radicals, leading to DNA damage and the activation of proteolytic enzymes that degrade dermal protein fibers. It also induces prolonged inflammation in the epidermis and dermis [13]. Consequently, UV radiation can cause skin photoaging and cutaneous carcinomas, as well as melanoma [14,15].

One of the aims of cosmetic products is to protect the skin against harmful environmental factors. The cosmetics industry is still searching for new, effective compounds that can protect the skin against UV radiation and the action of free radicals [16]. Several substances are known to help protect the skin and facilitate repair of damage caused by UV radiation and free radicals [17,18]. The application of folacin to the skin helps with skin cell regeneration and DNA repair and reduces the signs of photoaging [10]. However, folic acid can be decomposed by UV radiation. UV-induced folic acid photolysis causes folic acid to degrade in the body (in the blood and skin), resulting in deficiencies. Ultraviolet radiation causes photodegradation of folic acid to p-aminobenzoic-L-glutamic acid and 6-formylopterin, which is degraded to pterin-6-carboxylic acid [19,20,21,22,23,24]. 6-FPT can mediate sensitization reactions in the presence of oxygen and generate a variety of reactive oxygen species (ROS) [24]. ROS, generated under the influence of UV radiation, can activate various signaling pathways in cells, which may determine the death or survival of cells exposed to UV radiation [25]. The growth and survival of cells as well as the integrity of the genome are regulated by a complex network of pathways, in which cell cycle checkpoints, DNA repair, and programmed cell death have critical roles [26].

Cell cycle regulators are key factors for the control of proliferation and cell survival. In UV-radiation–exposed skin cells, P53 plays an active function as a transcriptional regulator [27]. The tumor suppressor protein P53 controls the cell cycle and apoptosis by transcriptionally activating pro-apoptotic proteins like BAX and sometimes downregulating anti-apoptotic proteins like BCL-2 [28]. When cellular stress is detected, P53 can induce cell cycle arrest, preventing cell division, or trigger apoptosis (programmed cell death). The balance between pro-apoptotic BAX and anti-apoptotic BCL-2 proteins is critical, with a higher BAX/BCL-2 ratio favoring cell death [29]. P53 is also a key factor inducing cell senescence, and in combination with other signaling pathways, it can lead cells to irreversibly arrest the cell cycle, which counteracts transformation of normal cells to cancer cells [30]. One of the key regulators of the cell cycle is cyclin D1 [31,32]. Cyclin D1 is an important regulator of cell cycle progression and can function as a transcriptional co-regulator. Cyclin D1 belongs to the family of serine/threonine protein kinases. Binding of cyclin D1 to kinases (the cyclin kinases 4 (CDK4) and 6 (CDK6) results in the formation of active cyclin–CDK complexes that phosphorylate protein that subsequently stimulate the expression of genes required for entry into S phase. Disturbed cyclin D1 gene expression alters cell cycle progression [33].

The aim of this study is to demonstrate the extent to which and the manner in which the use of folic acid as a UV protective agent is effective. The present study is conducted in an in vitro cell culture system. In our study, we employed electron paramagnetic resonance (EPR) to examine the impact of UV radiation on the interaction between folic acid and free radicals. Additionally, we employed molecular techniques to investigate the influence of UV radiation on the expression of genes that regulate the cell cycle: *CCND1*, *P53*, *BAX*, and *BCL-2* in cells protected by folic acid.

## 2. Results

### 2.1. Cell Viability

**Figure 1:**
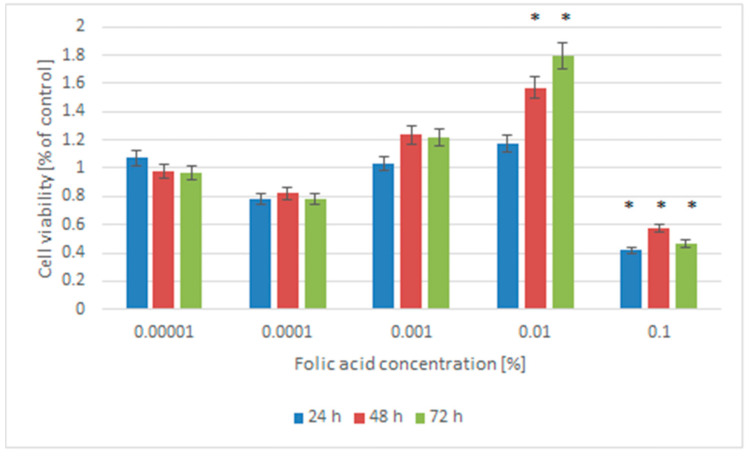
Influence of folic acid on the viability of NHDF-Ad cells after 24 h, 48 h, and 72 h of treatment. The results are expressed as a percentage of the untreated control cells (means ± SD; * *p* < 0.05 vs. control).

In our experimental model, we compared the effect of folic acid at several concentrations (0.1%; 0.01%; 0.001%; 0.0001%; 0.00001%) on the viability Normal Adult Human Dermal Fibroblasts (NHDF-Ad) to cells not treated with folic acid (0%) (Figure 1).

Folic acid at a concentration of 0.1% revealed a cytotoxic effect on the fibroblasts NHDF-Ad cell post hoc Tukey’s test compared to the control cell (untreated with folic acid). The viability of NHDF-Ad cells treated with 0.01% folic acid increased by more than 50% after 48 and 72 h compared to control NHDF-Ad cells. This increase in cell viability was statistically significant (48 h: *p* = 0.0233; 72 h: *p* = 0.0219; post hoc Tukey’s test). After 24 h, the viability of NHDF-Ad cells treated with 0.01% folic acid showed no significant difference compared to control NHDF-Ad cells (*p* > 0.05, *t*-test, post hoc Tukey’s) (Figure 1). Similarly, NHDF-Ad cells treated with 0.001%, 0.0001%, or 0.00001% folic acid showed no significant difference compared to the control group after 24, 48, or 72 h of treatment (Figure 1).

### 2.2. Viability Measurement of Cells Treated by Folic Acid and UV Irradiation

**Figure 2:**
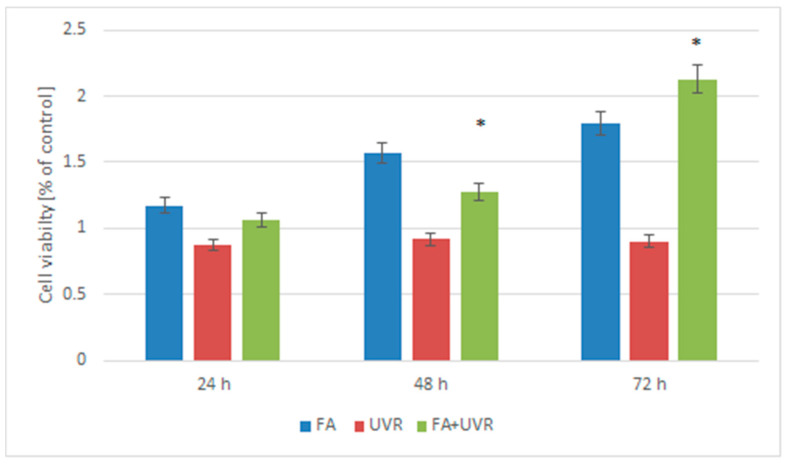
Viability of Normal Adult Human Dermal Fibroblasts (NHDF-Ad) treated with 0.01% folic acid (FA), UV radiation for 15 min (UVR), and 0.01% folic acid and UV radiation for 15 min (FA + UVR) after 24, 48 and 72 h. The results are expressed as a percentage of the control cells (means ± SD; * *p* < 0.05 vs. control).

The viability of NHDF-Ad cells treated with 0.01% folic acid and UV radiation for 15 min was assessed after 24 h, 48 h, and 72 h (Figure 2).

After 24 h, the viability of NHDF-Ad cells treated with 0.01% folic acid and UV radiation for 15 min showed no significant difference compared to control NHDF-Ad cells (*p* > 0.05, post hoc Tukey’s test). However, the viability of NHDF-Ad cells treated with 0.01% folic acid and UV radiation (15′) increased after 48 and 72 h, showing significant differences compared to control NHDF-Ad cells (48 h: more than 1.2-fold; 72 h: more than 2-fold) (*p* = 0.0214 and *p* = 0.0199; post hoc Tukey’s test).

### 2.3. Influence of UV Radiation on the mRNA Expression of Selected Genes That Regulate the Cell Cycle BAX, BCL-2, CCND1, and P53 in Cells Protected by Folic Acid

**Figure 3:**
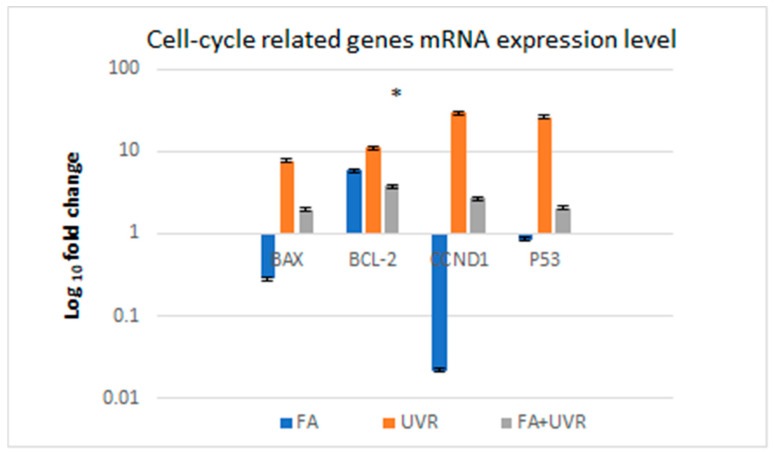
Expression of *BAX, BCL-2*, *CCND1*, and *P53* mRNAs in NHDF-Ad cells treated with 0.01% folic acid (FA), UV-radiation for 15 min (UVR), and 0.01% folic acid and UV-radiation for 15 min (FA + UVR). * *p* < 0.05 versus the control cells.

The regulation of the cell cycle plays an important role in maintaining genetic integrity. P53 can arrest human cells with damaged DNA in the G1 phase of the cell cycle, which allows the DNA to be repaired before the S phase. If DNA damage caused by UV radiation is severe and cannot be repaired, the apoptotic pathway is activated to eliminate the damaged cells. The protein P53, acting as a transcription factor, can induce apoptosis by increasing the expression of pro-apoptotic genes such as *BAX*. P53 also downregulates the expression of anti-apoptotic genes, such as *BCL-2* [34]. Cyclin D1 is an important regulator of the cell cycle and can function as a transcription co-regulator. Cyclin D1 belongs to the family of serine/threonine protein kinases. Binding of cyclin D1 to kinases (CDK4 and CDK6) results in the formation of active cyclin–CDK complexes that phosphorylate proteins, which subsequently stimulate the expression of genes required for entry into the S phase of the cell cycle. Disturbed cyclin D1 gene expression can alter cell cycle progression [35]. We, therefore, determined the expression levels of *BAX, BCL-2*, *CCND1*, and *P53* mRNA in the NHDF-Ad cells after treatment with 0.01% folic acid and UV radiation for 15 min. Exposure of NHDF-Ad cells to 0.01% folic acid and UV radiation for 15 min resulted in the up-expression of *BAX*, *BCL-2*, *CCND1*, and *P53* mRNA compared to control NHDF-Ad cells. The NHDF-Ad cells showed that folic acid at a concentration of 0.01% and UV radiation for 15 min increased the transcription of *BAX* and *P53* mRNA by approximately twofold, and the *CCND1* mRNA by two-and-a-half-fold. However, statistical analysis revealed no significant differences in the mRNA expression of the *BAX*, *CCND1*, and *P53* genes compared to the control cells (*p* > 0.05, post hoc Tukey’s test). The expression of the *BCL-2* mRNA gene in NHDF-Ad cells treated with folic acid at a concentration of 0.01% and UV radiation for 15 min revealed an increase of over threefold compared to the control cells. This increase in *BCL-2* mRNA expression was statistically significant (*p* = 0.014, post hoc Tukey’s test) (Figure 3).

### 2.4. The Effect of UV Radiation on the Interaction Between Folic Acid and Free Radicals Using Electron Paramagnetic Resonance (EPR)

**Figure 4:**
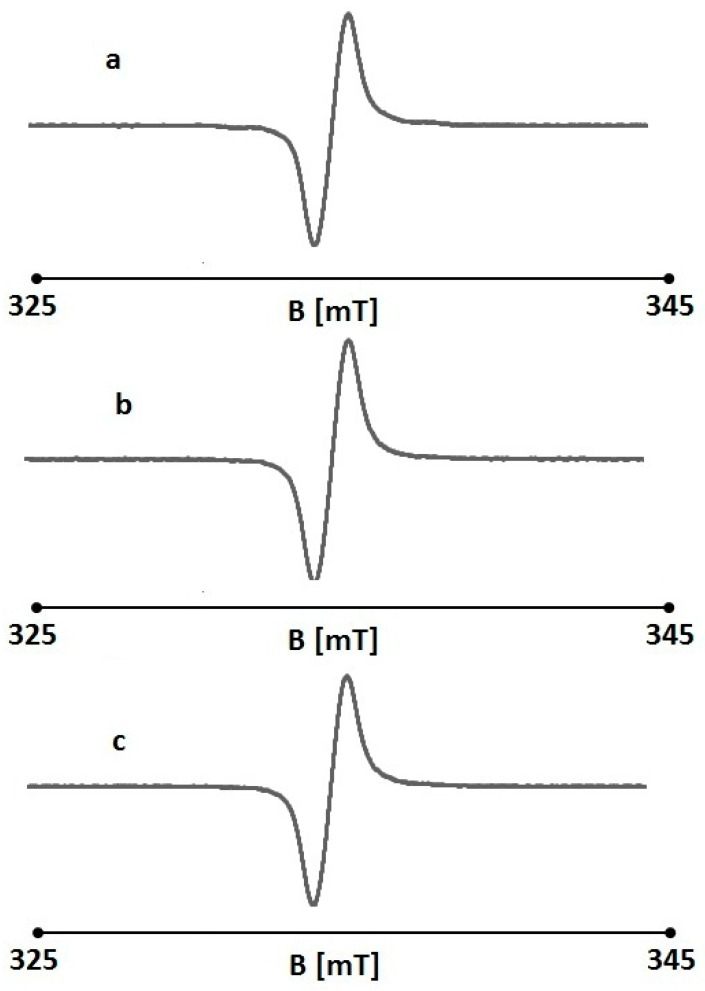
EPR spectra of DPPH with nonirradiated folic acid. The concentrations of folic acid: 0.1% (**a**), 0.01 (**b**), and 0.001% (**c**). B is the magnetic induction of the field produced by electromagnet of the spectrometer.

An EPR examination confirmed the antioxidant properties of non-irradiated folic acid. The EPR spectral parameters of the reference (DPPH in an ethanol solution) changed after the addition of folic acid. The amplitudes (A), linewidths (ΔB_pp_), g-factors, and asymmetry parameters A_1_/A_2_, and B_1_/B_2_ of the EPR lines of DPPH in ethanol solution and in ethanol solution together with nonirradiated folic acid are presented in Table 1. The exemplary EPR spectra of DPPH in ethanol solution with nonirradiated folic acid are shown in Figure 4. Folic acid interacts with the free radicals of the reference used—DPPH—and the result of these interactions is a decrease in the amplitude of the DPPH EPR line (Table 1). The quenching of the DPPH EPR line was similar for all the concentrations of folic acid used. However, at a concentration of 0.00001%, its interactions with free radicals were lower.

The broad EPR lines were observed for DPPH interacting with folic acid in ethanol, and the linewidths (ΔB_pp_) were in the range 0.45–0.48 mT (Table 1). These broad EPR lines indicate strong dipolar interactions between DPPH free radicals with unpaired electrons localized on nitrogen (N) atoms, which have characteristic g-values of 2.0036. Typical asymmetrical DPPH lines were measured, and the line shape parameters A1/A2 and B1/B2 strongly differed from 1 (Table 1).

**Figure 5:**
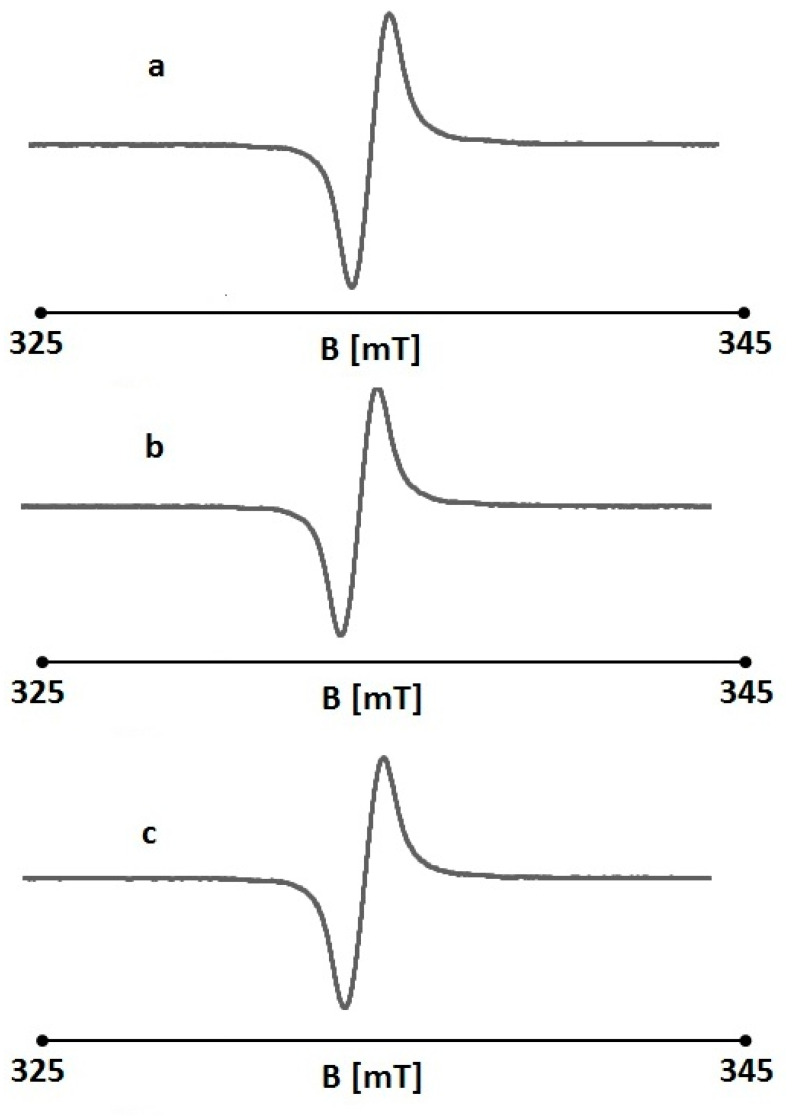
EPR spectra of DPPH with UV-irradiated folic acid. The concentrations of folic acid: 0.1% (**a**), 0.0% (**b**), and 0.001% (**c**). B is the magnetic induction of the field produced by electromagnet of the spectrometer.

The very interesting feature of folic acid was proven. The EPR results obtained for UV-irradiated folic acid are presented in Table 2. The exemplary EPR lines of DPPH interacting with UV-irradiated folic acid are shown in Figure 5. The amplitudes (A) of the DPPH lines increased after the addition of the irradiated samples. This indicates that folic acid lost its antioxidant properties during UV irradiation and did not interact with DPPH free radicals. The increase in the detected EPR line was probably caused by the free radical character of folic acid appearing after UV exposure.

The EPR spectra of DPPH in an ethanol solution with UV-irradiated folic acid (Figure 5) were similar to those of non-irradiated folic acid (Figure 4). They were strongly asymmetric, and the A1/A2 and B1/B2 parameters differed from 1 (Table 2). The EPR lines were dipolar broadened, with high linewidths in the range of 0.46–0.48 mT (Table 2). As expected, the typical g-factors of 2.0036 were obtained for the DPPH lines.

## 3. Discussion

The aim of our work was to investigate the photoprotective properties of folic acid in cells exposed to ultraviolet radiation in vitro. The effect of UV radiation on the expression of selected genes regulating the cell cycle in cells protected by folic acid was studied using normal human fibroblasts derived from adult skin (dermis). Fibroblasts were chosen for the study because they do not undergo as many programmed changes in normal human skin in vivo compared to keratinocytes. Under normal physiological conditions, the largest population of dermal fibroblasts are quiescent fibroblasts [36]. The major cell type of the epidermis is the epidermal keratinocyte, which undergoes a complex and carefully regulated process of keratinization. This process involves keratinocytes moving from the basal layer to the distinct outer layer, the *stratum corneum*, where they become specialized cells known as corneocytes [37]. Keratinocyte proliferation and differentiation are essential processes for epidermal stratification and *stratum corneum* formation [38]. Keratinocytes proliferate in the basal layer, where the stem cells also reside, and subsequently start their differentiation by changing their functional or phenotypic type. The keratinocyte differentiation programs are orchestrated by several transcription factors, signaling pathways, and epigenetic regulators [38,39]. The formation of corneocytes from epidermal keratinocytes represents a tissue-specific form of programmed cell death that differs from classical apoptosis because it does not result in fragmentation of the cell into apoptotic bodies and subsequent phagocytosis; however, the caspases and other elements of the molecular machinery of apoptosis may be involved in this process [40,41].

Human epidermal keratinocytes are constantly exposed to external stimuli that generate reactive oxygen species within the cells. Maintaining the redox state is key to preserving intracellular homeostasis. Originally, each cell type had its own defense systems against oxidative stress; thus, keratinocytes may have a unique system for regulating ROS levels [42]. Intracellular signaling reacts appropriately to changes in ROS level in cooperation with intra- and extra-cellular antioxidant agents, and is sometimes affected by excessive ROS generated by various stresses. Keratinocytes are more resistant to the lethal effects of UV light than fibroblasts [42,43]. We chose fibroblasts for our research because keratinocytes are protected from UVB damage and present a more efficient global genome repair system (GGR) than fibroblasts. Keratinocytes are more resistant to the lethal effects of UVB than fibroblasts, and *P53* plays a unique role in the UVB response of keratinocytes. D’Errico et al. revealed that keratinocytes are also more resistant than fibroblasts to the lethal effects of oxidizing agents (UVB radiation) and are characterized by a strong antioxidant capacity and higher susceptibility to ROS-induced apoptosis [44].

In our studies on the protective effect of folic acid on fibroblasts exposed to UV radiation, we used a monolayer (two-dimensional, 2D) cell culture model of normal fibroblasts derived from adult skin. Two-dimensional models of cell culture are simple, inexpensive, effective, and reliable models used to evaluate the effects of physical factors (e.g., UV radiation), chemical factors (pharmacologically active substances), as well as for understanding the relevant molecular and cellular mechanisms underlying various abnormalities and diseases (e.g., cancer) [45].

Monolayer models are still widely used in basic research at both the cellular and molecular levels. Despite their many advantages, these models are unable to reflect key aspects of in vivo conditions, since tissue function in vivo results from multiple cellular, biochemical, and spatial interactions. In vitro, in a monoculture, only the individual basic functions of the cells are expressed. Moreover, two-dimensional cultures do not provide conditions for interaction of cultured cells with other cell types, which is extremely important in the case of studies on the effects of UV radiation on the skin. In addition, 2D models also have other limitations, such as lack of tissue specificity, mechanical problems, biochemical disturbances, and incompatibilities between cells and the matrix [45,46].

Human skin is the largest organ, consisting of a stratified epidermis and an underlying dermis. Two-dimensional models (keratinocytes, fibroblasts, or melanocytes) do not reflect the relationship between cell layers and skin layers and, most importantly, do not allow for the topical application of cosmetic or pharmaceutical products. Three-dimensional (3D) constructs of the epidermis and full-thickness skin (epidermis and dermis) not only enable the study of skin barrier functions but also analyze intercellular interactions in the layers that make up the skin under various conditions (treatment of exogenous factors, chemicals, drugs). 3D skin models enable the study of interactions between cells and skin layers (epidermis and dermis) that affect the condition of the normal skin in response to exogenous factors. Currently, several 3D epidermis models (SkinEthic Rhe, Episkin, Lyon, France; Epiderm, MatTek, Ashland, OR, USA; Epidermal skin test 1000/EST1000, CellSystems Biotechnologie GmbH, Troisdorf, Germany) and full-thickness skin models (EpidermFT, MatTek, Ashland, OR, USA; StrataTest, StrataTech, Madison, WI, USA; Advanced skin test 2000/EST2000, CellSystems Biotechnologie GmbH, Troisdorf, Germany) are commercially available for not only testing potential new drugs but also for studying DNA damage, UV exposure, and wound healing [47].

In our study concerning the protective role of folic acid against the negative effects of ultraviolet radiation, we used a lamp that emitted both UVA and UVB radiation (Balance Facial Solarium GB 2000, Luxoplast Kunststofftechnik GmbH; Ampfing, Germany). The purpose of using a lamp emitting both UVA and UVB radiation was to replicate as closely as possible the conditions of natural UV radiation reaching the non-photoprotective skin. Ultraviolet (UV) radiation that reaches the Earth’s surface consists of UVA (320–380 nm) and UVB (290–320 nm) radiation [48]. Daily exposure to sunlight makes human skin and eyes the main targets of UV radiation. The UV components of solar radiation comprise about 95% UVA and 5% UVB—the amount of UVA radiation in sunlight is 20 times greater than UVB. Epidermal cells are targeted by both UVA and UVB radiation, while dermal cells and the extracellular matrix of the dermis are mainly targeted by UVA radiation. A total of 10% of UVB radiation and approximately 20% of UVA radiation of sun radiation reach the basal layer of the epidermis. The immediate responses of normal human skin exposed to UV radiation are UVB-induced erythema and pigmentation. Later changes include hyperkeratosis, acanthosis, the disorganization and misalignment of keratinocytes, dermal vascular ectasia, and mononuclear perivascular infiltration [49,50].

Both UVA and UVB radiation cause long-term effects on the skin, including premature aging/photoaging, immune suppression, and carcinogenesis. Moreover, UVA radiation is most often responsible for triggering phototoxic reactions, but UVB radiation and visible light can also cause phototoxicity [51]. The biological effects of UVA and UVB radiation are different, similar to the genes induced by these two ranges of solar radiation. Genes induced by the UV component of sunlight are generally grouped into either the UVA- or UVB-inducible category. This distinction is important because the differing mechanisms of DNA damage and cellular response contribute to varied cellular and tissue-level effects, including skin cancer development [52].

UVA generates reactive oxygen species and intermediates (lipid peroxidation—source of secondary free radicals*)*, which damage cellular targets (cell membranes, proteins, DNA). UVB radiation is not only oxidative but is also absorbed by DNA and proteins. The DNA-damaging properties of these wavelengths are due to direct absorption by target molecules, causing mutations, immune suppression, and carcinogenesis. UVA activation of genes is mediated by singlet oxygen, and this reactive oxygen species is involved in stimulating signaling cascades and transcription factor activation. UVB can generate other intermediates (e.g., hydrogen peroxide) that may be involved in signal transduction and gene activation. Nevertheless, certain genes (e.g., *MMP-1, collagenase 1*) can be induced by both UVA and UVB radiation [52].

Acute UV exposure causes extensive DNA damage that triggers DNA damage response pathways (DDR). DDR network is responsible for the maintenance of genome integrity in cells. The DDR proteins are divided into caretakers and gatekeepers. Caretakers protect the DNA of the genome by directly repairing DNA damage, while gatekeepers adapt DNA repair to the cell cycle or cell death. Caretakers and gatekeepers work together to maintain the integrity of the cell. Caretakers in DDR include damage sensors, signaling/mediator proteins, and effectors. Caretakers protect the genome against mutations, while gatekeepers induce cell death or cell cycle arrest of potentially tumorigenic cells [25,53,54]. The *P53* gene is the most well-known gatekeeper. UV-mediated DNA damage is detected by MRE11-RAD50-NBS1 (MRN) protein complexes (sensor of DNA damage). The formation of the MRN protein complex is intended to mediate the DNA damage signal to ATM kinase. Oxidation may also directly activate ATM, independently of MRN complex. ATM phosphorylates a variety of substrates following DNA damage, including P53-binding protein 1 (53BP1). Moreover, ATM activation leads to phosphorylation of downstream signal transducers, including serine/threonine-protein kinases CHK1/2 and P53, and results in subsequent inhibition of cyclin-dependent kinases (CDKs) that break the cell cycle in the G1-S, S, or G2-M phase of the cell cycle [55]. In the cellular response to UV-induced DNA damage, P53 is a key molecular mediator that acts as a transcriptional regulator. Low levels of UV radiation trigger transient P53 activation, with subsequent induction of cell cycle arrest, enabling DNA damage repair, whereas high levels of UV radiation lead to a more pronounced and sustained p53 activation, inducing apoptosis. Some signaling pathways, such as PI3K/AKT/mTOR (phosphoinositide 3-kinase/PI3K, serine/threonine protein kinase AKT, mammalian target of rapamycin/mTOR), can counteract P53 signaling by activating cell cycle transition, which may lead to hyperproliferation and neoplastic transformation. Alternatively, P53 may activate autophagy or induce premature cell senescence to prevent oncogenic transformation.

Therefore, altering the balance between these signaling pathways may determine the outcome between cell death and survival in UV-exposed cells. UV radiation also produces excessive amounts of reactive oxygen species (ROS), which can activate signaling pathways like mitogen-activated protein kinases (MAPKs) and/or phosphoinositide 3-kinase (PI3K/protein kinase B/AKT). Moreover, ROS activate transcription factors like nuclear factor erythroid 2–related factor 2 (Nrf2), hypoxia-inducible factor 1α (HIF-1α), activator protein 1 (AP-1), and nuclear factor kappa-light-chain-enhancer of activated B cells (NF-κB), which are associated with inflammation and carcinogenesis [25,56].

In phototherapy/photochemotherapy, strictly defined doses of radiation are used, e.g., UVA 1—low dose refers to 10–20 J/cm^2^ per single dose, medium dose UVA1 to 50–60 J/cm^2^ per single dose, and high dose UVA1 to 130 J/cm^2^ per single dose [57].

The Minimum Erythemal Dose (MED) is most commonly used to study the effects of radiation on the skin. MED is widely used as a biological unit of exposure dose in clinical and experimental photodermatology and is based on a single acute exposure to UV radiation. Erythema (inflammation) is the most visible clinical symptom of UV exposure. UVB radiation is mainly responsible for the development of erythema, and individual sensitivity to UV radiation can be determined by measuring MED. MED is defined as the UV radiation dose (J/m^2^) in a given spectrum that causes noticeable reddening of the skin. A standard erythema dose (SED) is equivalent to an erythemally effective radiant exposure of 100 J/cm^2^. About three SEDs are required to produce a just-perceptible MED in the unacclimatized white skin of the most common northern European skin types (III/IV skin phototype according to Fitzpatrick’s classification). Exposure to UVB radiation is divided into several types: acute, subacute, and chronic. Repeated daily suberythemal exposure (0.25 MED) causes clinically visible erythema after 2–3 exposures, especially in sun-sensitive skin phototypes I and II, but to a lesser extent in phototypes III/IV [58]. MED has been defined as the lowest radiant exposure to UVR that is sufficient to produce erythema, with sharp margins 24 h after exposure. MED is used as a unit of “exposure dose,” and the representative value for sun-sensitive individuals is 200–250 J/m^2^ (MED depends on skin phototype in the Fitzpatrick classification) [59].

Within Europe, sun tanning products must conform to a revised standard EN 60335-2-27:2013. This standard classifies UVR emitters into four types according to different limits of the effective irradiance in two different wavelength-bands and total effectiveness. The international standard IEC 60335-2-27:2015 in its consolidated version regulates parameters of sun tanning appliances. Appliances shall have effective irradiances limited as follows: a total effective irradiance not exceeding 300 mW/m^2^; the total wavelength-band related effective irradiance not exceeding—150 mW/m^2^ for wavelengths 250–320 nm and 320–400 nm. Erythema effective irradiance is determined over UVA and UVB wavelength regions 250 nm–320 nm (UVB) and 320 nm–400 nm (UVA). Erythema is the reddening of the skin, which is an inflammatory response caused by, for example, the actinic effect of exposure to UV radiation. Erythemal effective irradiance is measured over the wavelength range 250 nm to 400 nm [60].

Balance Facial Solarium GB 2000 (Luxoplast Kunststofftechnik GmbH; Ampfing, Germany) is a type 3 UV appliance suitable for household use without training. UV appliance type 3 is characterized by <0.15 W/m^2^ (150 mW/m^2^ for wavelengths 250–320 nm and 320–400 nm, respectively) erythemal effective irradiance measured both in 250 nm–320 nm and 320 nm–400 nm. Our study used 15 min of UVA/UVB radiation exposure, which gives a value of 135 J/m^2^. The maximum time that people of different skin phototypes can expose untanned and unprotected skin to the sun during the day without sunburn is 10 min for phototype I, 20 min for phototype II, and as much as 60 min for phototypes V and/or VI [61].

UVB-generated ROS activate the MAPK pathway in skin cells, activating various transcription factors (i.e., AP-1, NF-κB) and finally inducing skin photodamage, involving chronic inflammation, elastosis (accumulation of abnormal elastin fibers), and degradation of extracellular matrix components by metalloproteinases (MMPs). Pivotal role in the degradation of skin collagens play AP-1 transcription factor. Collagen is very sensitive to UVB radiation; even a suberythemogenic dose of UVB (less than 0.1 MED) can stimulate collagen proteolysis. The MAPK pathway is also a key element in UVB-induced inflammation. ROS generated by UV radiation may cause the activation of NF-κB, the family of transcription factors. The most important function of these transcription factors is to regulate UVB-induced inflammation, immune response, cell proliferation, and differentiation. Moreover, UVB irradiation results in the appearance of apoptotic cells (called sunburn cells) in the epidermis. UVA radiation, reaching the dermis, causes ROS formation. Oxidative stress is responsible for numerous damages to cellular structures (lipid peroxidation, DNA damage, protein carbonylation) and activation of downstream intracellular signaling pathways. These cellular and molecular changes induce mutations, apoptosis, remodeling of the dermis, inflammatory reactions, and abnormal immune responses. UVA radiation induces changes in gene expression in skin fibroblasts, supporting the thesis that skin fibroblasts are sensitive to UVA radiation. Ultraviolet radiation not only causes photoaging but also contributes to photo-immunosuppression in skin cells, which may contribute to the development of skin cancer and melanoma [62].

Folic acid plays a key role in the one-carbon cycle, which is essential for DNA synthesis, repair, and methylation [9], and exerts its cellular functions by binding to and activating folate receptors (FRs). Folic acid receptors (FRs) are cell-surface proteins that bind folate with high affinity and mediate its uptake into cells [63,64,65]. Four isoforms of FRs have been identified: FR α, FR β, FR δ, and FR γ. FR α is the most common isoform [63] and has a high affinity for folates not present in a normal balanced diet, such as folic acid [66]. Normal cells have limited folate receptor α (FRα) expression, but this can increase when folate levels are low or when elevated folate levels are needed for cell growth and DNA repair [63].

As eukaryotic cells cannot synthesize folic acid themselves, it must be delivered to them either via the reduced folate carrier, which is present in all cell types, or via the FR, which is only expressed in certain cells. FRs are cell surface glycoproteins with a high affinity for folic acid and are attached to the plasma membrane by a glycosylphosphatidylinositol (GPI) anchor. FR α is the most common isoform and has a higher affinity for folic acid, while FR β is mainly found in some cells activated macrophages. The expression and function of FR δ remain unclear, as it has not yet been detected in normal human tissue [63].

FR α and FR β are membrane-associated proteins, whereas FR γ is a secreted protein due to the absence of a signal peptide for a GPI anchor at its C-terminus. The expression of FRs on cells in different tissues depends on the types of FR isoforms present. FR α is expressed in the epithelial cells of normal tissues, such as type I and II alveolar cells in the lungs, ovary, fallopian tube, and uterus. Moreover, FR α is overexpressed in many malignant tumor cells of epithelial origin, including lung, ovarian, cervical, endometrial, brain, and breast cancers. FR β is expressed in neutrophils, CD34^+^ hematopoietic progenitor cells, placenta, spleen, and thymus. Expression of FR β is also found in pathogenic cells, such as acute myelogenous leukemia (AML) cells and chronic myelogenous leukemia (CML) cells. FR β is highly expressed on activated, but not normal and resting macrophages, which are implicated in the pathogenesis of human inflammatory diseases, such as rheumatoid arthritis, psoriasis, Crohn’s disease, systemic lupus erythematosus, atherosclerosis, diabetes, ulcerative colitis, osteoarthritis, glomerulonephritis, and sarcoidosis [67]. There is little or no expression of functional folate receptors on lymphocytes, B cells, mast cells, or fibroblasts [68].

However, folic acid receptor (FR)-targeted cancer therapy is an approach that uses the high expression of FRs on cancer cells, particularly the FR α isoform, to selectively deliver therapeutic agents directly to tumors [63,69].

There are no published data on the effect of UV radiation on the expression of folate receptors. Presumably, UV radiation, by causing photodegradation of folate, reduces its concentration. Reduced folate levels may result in increased receptor expression in normal cells. However, this issue requires further study.

Standard cell culture media contain 2200 nM (RPMI) or 9000 nM (DMEM) of the non-physiological, synthetic folic acid [40,70]. In cell culture studies, cells and tissues are subjected to elevated levels of folates that greatly exceed the physiological levels found in tissues to support growth and proliferation. Our results seem to confirm these findings, as we demonstrated increased viability in normal human dermal fibroblasts treated with 0.01% folic acid after 48 h and after 72 h (statistically significant) and treated 0.001% folic acid after 48 h and 72 h (statistically non-significant) compared to control NHDF-Ad cells. Other experimental data have shown that folic acid at a concentration of 0.01% improves the viability of the human primary fibroblasts and stimulates their proliferation [10]. Furthermore, Dębowska et al. demonstrated increased viability of fibroblasts exposed to folacin compared to control cells after UVB radiation exposure. The survival rate of cells cultured in the presence of folic acid after exposure to UVB radiation was almost twice as high as the survival rate of control cells (not treated with folacin prior to UV exposure) [10]. UVB (290–320 nm) radiation accounts for approximately 5% of natural ultraviolet radiation reaching the Earth; the remaining 95% is UVA radiation. Both types of UV radiation are associated with the development of cancer. UVA has also been reported to induce senescence in skin fibroblasts thus playing a key role in dermal photoaging. Moreover, UVA radiation penetrates the dermis more effectively, affecting the cells and extracellular matrix of the dermis [71]. The main negative effects of UV radiation on cell DNA include the formation of various photoproducts, the generation of free radicals, and subsequent strand breaks. The main photoproducts formed during exposure to UV radiation are cyclobutanopyrimidine dimers (CPDs) and 6-4 photoproducts (6-4PP) as well as Dewar valence isomers. UV radiation can also indirectly cause DNA damage by producing singlet oxygen or free radicals in photodynamic reactions. One of the most reactive forms of oxygen (ROS) is the hydroxyl radical (OH*), which has the ability to damage DNA [55].

In addition to these photoproducts, UV radiation can induce single- and double-strand breaks in DNA, leading to cellular damage and genetic aberrations [72,73,74]. The clonal expansion of cells with DNA damage is a key stage in tumor formation, and the burden of cellular mutations correlates with the risk of malignant transformation. Driver genes, such as 53, play a pivotal role in controlling the cell cycle [75] and act as the key molecular mediators in the response of cells to UV radiation. In skin cells exposed to UV radiation, P53 plays an active role as a transcription regulator. Low doses/levels of UV radiation cause transient activation of P53 and then induce cell cycle arrest, allowing DNA damage to be repaired. In contrast, high doses/levels of UV radiation lead to prolonged activation of P53, which can induce apoptosis. However, P53 can activate autophagy or induce premature cell aging to prevent malignant transformation. UV-activated P53 promotes apoptosis by activating the transcription of proapoptotic genes such as P53-upregulated modulator of apoptosis (*PUMA*), and phorbol-12-myristate-13-acetate-induced protein 1 (*PMAIP1*), which bind to and inhibit pro-survival BCL-2 proteins such as BCL-2 and BCL-XL, thereby releasing proapoptotic effector proteins BCL-2-associated X protein (BAX), BCL-2 Antagonist/Killer (BAK), and BCL-2 antagonist of cell death (BAD) [25].

UV exposure can cause the activation of epidermal growth factor (EGF) and IL-1 receptors, which may contribute to the activation of the JNK kinase cascade within minutes of UV exposure [76]. Rehemtula et al. revealed that UV radiation may induce apoptosis by the activation of cell surface death receptors CD-95 (Fas/APO-1) [77]. Moreover, UV radiation may activate the TNF receptor-1, TNF-α–related apoptosis-inducing ligand (TRAIL) receptors, and death receptors 3, 4, and 5 (DR-3, DR-4, and DR-5) [78]. Moreover, UV radiation triggers the activation of MAPK signaling pathways, which are involved in regulating cell proliferation, differentiation, apoptosis, and tumorigenesis [79]. The balance between the expression of *P53* and the expression of *BCL-2* is a key point for determining apoptosis to UV radiation [80], and P53 can interact with the mitochondria-mediated pathway and BCL-2 and BCL-xL proteins to regulate apoptosis [79]. Oxidative stress may play a central role in the regulation of apoptosis, too. BCL-2 protein may function as an antioxidant, regulating levels of reactive oxygen species and controlling early entry into apoptosis [80]. Cells expressing BCL-2 are inherently resistant to additional oxidative stress without directly reducing ROS levels. However, BCL-2 itself can reduce ROS levels within cells. BCL-2 increases cellular glutathione levels, alters the intracellular distribution of reduced glutathione, or directly binds to glutathione. However, BCL-2 does not appear to replace glutathione, as BCL-2-mediated survival can be reversed if reduced glutathione is sufficiently depleted in an environment lacking cysteine and methionine. Furthermore, BCL-2 inhibits hyperglycemia-induced lipid peroxidation and the formation of advanced glycation end products (AGEs) without altering ROS levels. BCL-2 increases the activity of the membrane Na+/K+-ATPase, a redox-sensitive ion pump, in unstimulated cells. However, BCL-2 can increase ROS levels not only in glutathione-depleted cells but also in fibroblasts. The BCL-2-dependent increase in ROS levels may cause a modest increase in cellular oxidative stress, leading to increased cellular antioxidant capacity and improved resistance to additional oxidative stress. These effects may also be related to BCL-2 expression level. BCL-2 may also improve mitochondrial function by reducing chronic oxidative stress, and this antioxidant effect is most likely indirect and involves modulation of mitochondrial bioenergetics, i.e., substrate fuel availability, electron flow, leakiness of the inner mitochondrial membrane, or conjugation with adenine nucleotides to reduce ROS in mitochondria [81]. Increased *BCL-2* expression can occur in response to oxidative stress, potentially serving as a protective mechanism by facilitating DNA repair and increasing cellular antioxidant defenses [82,83,84].

In our experiment, the viability of cells treated with 0.01% folic acid and exposed to 15 min on UV radiation was reduced, compared to that of cells treated only with 0.01% folic acid after 48 h. However, the viability of cells treated with 0.01% folic acid and exposed to 15 min of UV radiation increased, compared to cells treated with 0.01% folic acid or exposed to 15 min of UV radiation, administered separately after 72 h. Therefore, the viability of cells treated with 0.01% folic acid and exposed to 15 min of UV radiation estimated at 48 and 72 h, shows significant differences. These differences may be due to the activation of different cell signaling pathways to UVA or/and UVB radiation. UVB is absorbed by aromatic heterocyclic bases, resulting in the formation of cyclobutane pyrimidine dimers (CPDs) and pyrimidine (6-4) and pyrimidone (6-4PP) photoproducts. Uncorrected errors can potently inhibit DNA and RNA polymerases, causing mutagenesis or cell death. UVA induces the formation of ROS through interaction with endogenous photosensitizers, which can consequently lead to damage to cell membranes, DNA, and proteins through oxidative reactions [25]. Studies in cell lines indicate a wide array of transcriptional changes in response to UV irradiation. The number of genes involved in cell cycle inhibition and progression were upregulated following UV treatment [55,85].

UV-induced DNA damage activates various pathways, leading to dissociation of the p53/MDM2 (mouse double minute 2) inhibitory complex, and two major kinases, ataxia telangiectasia mutated (ATM) and ataxia telangiectasia-related and Rad3 (ATR), mediate phosphorylation of P53, preventing its degradation. ATM is a key kinase involved in the DNA damage response (DDR) pathway. When DNA is damaged, ATM is activated and initiates a signaling process that coordinates the repair of this damage or, if the damage is too severe, leads to cell death. Mild DNA damage leads to ATM activation, which can directly phosphorylate P53 at Ser15 and mediate P53 Ser20 phosphorylation via activation of its direct substrate checkpoint kinase 2, CHK2. Furthermore, phosphorylation of serine 15 and serine 20 in P53 disrupts its degradation and, thus, promotes P53 stabilization and subsequent transactivation of its target gene [25].

ATM activation leads to phosphorylation of downstream signal transducers, including serine/threonine-protein kinases CHK1/2 and P53 and results in subsequent inhibition of cyclin-dependent kinases (CDKs) that break the cell cycle in the G1-S, S, or G2-M phase of the cell cycle [55].

When UV-induced DNA damage occurs before entry into S phase, pP53 arrests the cell cycle in G1 phase, in part through transcriptional induction of cyclin-dependent kinase inhibitor 1A (CDKN1A/P21/WAF1/CIP1), a potent inhibitor of several cyclin-dependent kinase complexes. P21 can also block the elongation step of DNA replication in the S phase of the cell cycle or can inhibit cyclin-dependent kinase complexes with both cyclin A and cyclin B, promoting G2 arrest. The main coordinator of replication is proliferating cell nuclear antigen (PCNA), which is also a P53 transcriptional target. In the cellular response to UV-induced DNA damage, P53, a key molecular mediator, acts as a transcriptional regulator. In response to UV damage, P53 acts as a transcription factor that regulates the expression of various genes, including cyclin-dependent kinase inhibitor 1A (*P21*) and the pro-apoptotic (BAX) and *BCL-2*-binding component 3 (*PUMA*) proteins. PUMA works as an apoptosis inducer because of the higher affinity of binding toward anti-apoptotic proteins. P21 (up-regulated in a P53-mediated gene expression) form complexes with cyclin-CDK leading to cell cycle arrest. Furthermore, P21 inhibits CDKs, preventing the phosphorylation of the retinoblastoma-associated protein (PRB), thereby disabling the G1 to the S transition of the cell cycle. Gadd45α (growth-arrest and DNA damage-inducible) is also a P53 effector involved in the control of the transition from G2 to M phase of the cell cycle [25].

P53 plays transcription-dependent and -independent functions during cell cycle arrest, during which DNA repair mechanisms (nucleotide excision repair NER and base excision repair BER) are activated. The additional phosphorylation serine 46 in pP53 results in preferential transactivation of cell death-stimulating pP53 target genes and P53-dependent induction of mitochondrial outer-membrane permeabilization, involving anti- and pro-apoptotic members of the B-cell lymphoma 2 (BCL-2) family [25].

BAX protein with BCL-2 homologous antagonist/killer (BAK) is responsible for the activation of the intrinsic apoptotic cell death. The activity of BAX and BAK is restricted by anti-apoptotic proteins such as apoptosis regulator (BCL-2) or BCL-2-like protein 1 (BCL-Xl) [55].

Moreover, the intracellular ratio of BAX/BCL-2 is considered as the key indicator in the decision of the cells to respond to apoptotic signals. A cell with a high BAX/BCL-2 ratio will be more sensitive to the given apoptotic signal as compared to a similar cell type with a low BAX/BCL-2 ratio [86]. Cyclin D1 is an important regulator of cell cycle progression and can function as a transcriptional co-regulator. Disturbed cyclin D1 gene expression alters cell cycle progression. In our study, we investigated the influence of UV radiation on the expression of genes that regulate the cell cycle, *CCND1*, *P53*, *BAX*, and *BCL-2*, in normal human dermal fibroblast derived from adult skin protected by folic acid. Exposure of NHDF-Ad to folic acid at concentration 0.01% and UV-radiation for 15 min resulted in up-expression of *BAX*, *BCL-2*, *CCND1*, and *P53* mRNAs compared to control NHDF-Ad cells. Expression of mRNA *BCL-2* gene in NHDF-Ad cells treated to folic acid at concentration 0.01% and UV-radiation for 15 min revealed over three-fold increase, compared to control cell, and revealed statistically significant differences. The *BAX*/*BCL-2* ratio in cells treated with folic acid and exposed to UV radiation (0.7) also decreased, compared to the *BAX*/*BCL-2* ratio (0.5) in cells exposed only to UV radiation and not treated by folic acid. Cyclin D1 is mostly known as a regulator of cell cycle progression. Cyclin D1 modulates the transition from the G1 to the S phase [87]. In human keratinocyte cell line, both nonlethal UVA and low-level UVB exposure were shown to activate AKT, with resultant cyclin D1 upregulation and G1 to S cell cycle transition [88]. In our study, analysis of the viability of cells exposed to folic acid and UV irradiation confirmed that the cells not only survived but also proliferated. These results may suggest that folic acid does indeed protect cells from the negative effects of UV radiation. In addition, the cells were exposed to UV radiation for 15 min, which seems to be not cytotoxic. The World Health Organization (WHO) recommends 5 to 15 min of sun exposure 2 to 3 times a week [89]. Furthermore, 10–15 min of exposure to ultraviolet radiation (without using sunscreen) is sufficient for vitamin D synthesis [90].

Exposure of an aqueous solution of folic acid to UV radiation causes its decomposition, forming 2-amino-3,4-dihydro-4-oxo-6-pteridinecarboxaldehyde or 6-formylopterin (6-FPT), which then yields pteridin-6 -carboxylic acid (PCA or 6-carboxyptyrin). The degradation of folic acid under the influence of UV light depends on various parameters, mainly light, temperature, concentration, oxygen, pH, singlet oxygen, and electron beams. 6-FPT is the main photoproduct of FA, formed as a result of FA degradation in human skin and blood under the influence of ultraviolet radiation. Ultraviolet B (UVB) radiation is more effective in degrading FA than UVA radiation. The photoconversion of FA to 6-FPT requires the presence of oxygen and is accompanied by the formation of hydrogen peroxide (H_2_O_2_).

6-FPT can cause sensitizing reactions in the presence of oxygen, known as photodynamic effects. Oxygen-dependent sensitizing reactions are divided into two types, I and II, which involve unstable molecules. 6-FPT reacts with NADH in the presence of O_2_ to generate a variety of oxygen species. Excited 6-FPT can oxidize DNA components [24].The formation of 6-FPT leads to the production of ROS in cells. Depending on the cell type and conditions, the formation of ROS associated with the photodegradation of folic acid and the formation of 6-FPT may lead to cell protection or cell damage by free radicals. Ishii et al. showed that intracellular ROS generated by 6-formylpterin decline the intracellular redox state to an oxidant state, which suppresses caspase activity and prevents the apoptotic cell injury of hepatocytes [91]. Additionally, Funakoshi et al. showed that 6FP protects retinal neurons from transient ischemia–reperfusion injury, at least in part by inhibiting apoptotic cell death [92]. Photoproducts formed upon UV irradiation of folic acid can damage nuclear DNA, stimulate nuclear DNA repair, and alter the expression of key genes involved in the cell cycle, thereby promoting cell death or survival [25,55,85]. However, UV radiation induces mitochondrial and cytosolic ROS production and induces mitochondrial changes [93].

In our studies, we determined the viability of cells treated with 0.01% folic acid and exposed to 15 min of UV radiation, indirectly using the WST-1 assay. The principle of the WST-1 assay is the conversion of stable tetrazolium salt (WST-1) into a water-soluble formazan dye by mitochondrial enzymes (mainly dehydrogenases) present in living cells. The absorbance value of the formazan product is directly related to the number of metabolically active cells in cell culture. Therefore, the assessment of the viability of cells treated with 0.01% folic acid and exposed to UV radiation (15 min), assayed at 48 and 72 h, showed differences, which may be due to the indirect assessment of the number of metabolically active cells exhibiting mitochondrial enzymes activity (WST-1 assay). It should be remembered that cells activate repair mechanisms in response to damage caused by UV radiation, for example, DNA damage. Seventy-two hours after exposure, the results of cell activity utilizing such repair systems may already be evident, leading to increased metabolic activity of the cells themselves.

Assays directly assessing the number of viable cells by measuring DNA (e.g., BrdU incorporation, DNA-binding dyes CyQuant) would be preferable and would allow for more precise determination of cell numbers at specific time points.

It has been shown that 6-FPT in normal cells has a protective effect against free radical damage to cells, while in cancer cells, 6-FPT may enhance oxidative stress and increase the activity of anticancer drugs [24]. We employed electron paramagnetic resonance (EPR) to examine the impact of UV radiation on the interaction between folic acid and free radicals. Electron paramagnetic resonance (EPR) (electron spin resonance, ESR) is one of the methods used to measure oxidative stress by direct detection of unpaired electrons. EPR involves the detection of electron spin excitation in an applied magnetic field [94]. The 2,2-diphenyl-1-picrylhydrazyl radical (DPPH•) is a neutral, stable radical used to assess the activity of antioxidants [95].

The amplitude (A) of the DPPH lines increased following the addition of irradiated folic acid samples. This indicates that folic acid lost its antioxidant properties during UV irradiation and did not interact with the DPPH free radicals. The increase in the detected EPR line was probably caused by the free radical character of folic acid appearing after UV exposure. In our research, it would be worthwhile to confirm not only the presence of UV degradation photoproducts of folic acid using high performance liquid chromatography (HPLC), thin-layer chromatography (TLC), high-resolution mass spectrometry *(*HRMS*)*, UV spectrophotometry, or other advanced research methods, but also to assess the effect of a precisely defined amount of these photoproducts on cells and its protective/destructive role.

High performance liquid chromatography (HPLC), thin-layer chromatography (TLC), and UV spectrophotometry are the most commonly used methods to evaluate folic acid photodegradation products [21,22,96,97]. HPLC is an advanced analytical technique employed to separate, identify, and quantify components in complex mixtures [98].

Akhtar et al. revealed that the analysis of folic acid and its photoproducts using the HPLC clearly demonstrates the separation of folic acid and its photodegradation products, i.e., pterine-6-carboxylic acid and *p*aminobenzoyl-L-glutamic acid. However, some deviations were observed in the kinetics of folic acid photodegradation. These deviations may be due to factors such as minor changes in light intensity during photolysis, the sensitivity of the compound to HPLC detection at different reaction stages, and variations in column efficiency and mobile phase pH [96].

Thin layer chromatography (TLC) is an affinity-based method used to separate compounds in a mixture. TLC is a highly versatile separation method that is widely used for both qualitative and quantitative sample analysis. Quantitative TLC (qTLC) analysis involves developing spots on a TLC plate and then visualizing them for detection using methods such as densitometry, image analysis using software, or scraping the separated compounds from the plate for extraction and spectrophotometric analysis or mass spectrometry [99]. High-resolution mass spectrometry (HRMS) is defined as a technique that combines high-performance liquid chromatography (HPLC) with high-resolution time-of-flight mass spectrometry (LC-TOF-MS) to identify and characterize unknown substances by comparing their mass spectra to a reference library. Goossens et al. investigated the properties of photodegradation product of folic acid, 6-formylpterin (6-FPT), and highlighted its capacity to form covalent adducts with the ROS-scavenging drug edaravone using HRMS [24]. Not only is HRMS suited to identifying unknown substances, but it can also be used for reliable quantitative purposes [100].

Off et al. applied spectroscopic methods to study folic acid photodegradation. Folic acid consists of three molecular parts: pterin, aminobenzoyl and glutamate. Bond cleavage between C9 and N10 was reported for UVA- and UVB-exposed FA. This leads to changes in the spectral characteristics of the UV-radiated solution of folic acid [23]. These changes in spectral characteristics may be measured by UV/VIS spectrophotometry. Even though folic acid has a low fluorescence quantum yield, when the molecule is exposed to UV radiation, the bond between 6-methylpterin and p-aminobenzoic acid is broken, and the fluorescence of the solution increases. The fluorescence of folic acid photodegradation products can be measured using fluorescence spectroscopy [101].

EPR examination confirmed the antioxidant properties of nonirradiated folic acid. A few literature data suggest that folates may act as antioxidants [102,103].

Antioxidant activity and the level of oxidative damage in cells can be estimated indirectly by using biomarkers, because reactive oxygen species are compounds that are difficult to measure when assessing oxidative stress, due to their very short half-life. However, when ROS bind to a specific biological molecule, the result is the formation of chemically modified molecules. Many of these molecules are used as biomarkers.

Elevated levels of free radicals may lead to the modification of lipids, proteins, nucleic acids, and other compounds. The most important mechanisms of protection against free radicals are intracellular. These mechanisms include the action of enzymatic antioxidants such as catalase (CAT), superoxide dismutase (SOD), and glutathione peroxidases (GPx). Changes in the activity of these enzymes may indicate the antioxidant status of cells. Antioxidant status can also be assessed using non-protein antioxidants such as glutathione, coenzyme Q (CoQ10, ubiquinone, 1,4-benzoquinone), and alpha-lipoic acid. Lipid peroxidation is a free radical chain reaction. The oxidation of unsaturated fatty acids or other lipids results in peroxide compounds (called secondary free radicals). Lipid peroxidation biomarkers include malondialdehyde (MDA), thiobarbituric acid-reactive substances (TBARS), and acrolein (ACR). Protein peroxidation biomarkers include protein carbonyls, di-tyrosine/bi-tyrosine (DiY/DiT), and m-tyrosine/o-tyrosine. Among the major products of DNA oxidation are 8-hydroxy-20-deoxyguanosine (8-OHdG) and 8-oxoguanine (8-oxo-Gua). These products are the most commonly used biomarkers of oxidative DNA damage [104].

Exposing skin cells (fibroblasts) that have been treated with folic acid for several days or several dozen days to UV radiation and assessing biomarkers of oxidative damage to lipids, proteins, and DNA would help to explain the possible mechanisms underlying the photoprotective effect of folic acid. It would also allow us to exclude the potential destructive effect of folic acid photodegradation products on cells.

The use of antioxidants can result in maintaining a pro- and antioxidant balance in cells, tissues, and organs. Pro-oxidative status is a critical driver in the pathogenesis and progression of many chronic diseases, including cancer and skin photoaging. Antioxidants, such as ascorbic acid (vitamin C), α-tocopherol (vitamin E), folate, β-carotene, ubiquinone (coenzyme Q10), bioflavonoids, selenium, and folic acid have beneficial effects in ameliorating oxidative stress [105,106]. Folic acid exhibits protective properties in fibroblasts in vitro against the negative effects of UV radiation.

## 4. Materials and Methods

### 4.1. Cell Source and Culture

The effect of UV radiation on the expression of selected genes regulating the cell cycle in cells protected by folic acid was studied using normal human fibroblasts derived from adult skin (dermis). Normal Adult Human Dermal Fibroblasts (NHDF-Ad) were obtained from the tissue culture collection of Clonetics (CC-2511, Lonza Walkersville Inc., Walkersville, MD, USA). Cells were cultured in Dulbecco’s Modified Eagle’s Medium (DMEM), supplemented with 4 mM L glutamine, 4500 mg/L glucose, 1 mM sodium pyruvate, and 1500 mg/L sodium bicarbonate (ATCC American Type Culture Collection, Manassas, VA, USA) and supplemented with 10% fetal bovine serum (FBS, American Type Culture Collection, Manassas, VA, USA) and 1% pen/strep solution (100 U/mL penicillin, 200 μg/mL streptomycin) (Sigma Aldrich, St. Louis, MO, USA). Cells were grown in monolayers to confluence in Nunc Easy Flasks, with filter 25 cm2 culture surfaces (T25, Thermo Scientific Nunc, Roskilde, Denmark). Cells were grown at 37 °C and 5% CO_2_ in a humidified incubator. At confluence, cells were routinely passaged with a trypsin 0.25% EDTA solution (1×) (Sigma Aldrich; St. Louis, MO, USA).

### 4.2. Folic Acid (FA)

Folic acid (F8758, Sigma) used in the study was purchased from Sigma Aldrich^®^ (cat. no. F8758, St. Louis, MO, USA) and dissolved in 1 M NaOH according to the manufacturer’s procedure. An aliquot of a 1% (22.6 mM) stock solution of folic acid was stored in the dark at −70 °C, thawed, and diluted with cell culture medium to the appropriate concentration before use. The final concentration in the experimental wells did not exceed 0.1% (*v*/*v*).

### 4.3. Cell Viability Measurement

The cytotoxic effect of folic acid was determined by WST-1 test (Roche Diagnostics GmbH, Mannheim, Germany) according to the manufacturer’s protocol. In brief, Normal Adult Human Dermal Fibroblasts (NHDF-Ad) were seeded in 96-well plates at a density of 5 × 10^4^ cells/well. Plates were incubated at 37 °C in 5% CO_2_ for 24 h to allow attachment to the bottom of the wells. After 24 h, the medium was changed to medium supplemented with folic acid at the following concentrations: 0% (control), 0.1%; 0.01%; 0.001%; 0.001%, and 0.00001%. The cells were treated with folic acid for 24, 48, and 72 h.

After 24, 48, and 72 h exposure at 37 °C in 5% CO_2_, the medium containing folic acid from each well was replaced with fresh medium (100 μL), and 10 μL of WST-1 test (Roche Diagnostics GmbH; Mannheim, Germany) was added to each well. During the 45 min incubation period, any viable cells converted the stable tetrazolium salt (WST-1) into a water-soluble formazan dye. The absorbance value of the formazan product is directly related to the number of metabolically active cells in the cell culture. After 45 min of incubation, the absorbance of the water-soluble formazan dye in each well was measured using a microplate reader UMV340 (Biogenet Asys Hitech GmbH, Eugendorf, Austria) at 450 nm. All concentrations of folic acid were tested in triplicate, and the assay was carried out in three separate experiments.

### 4.4. Viability Measurement of Cells Treated by Folic Acid and UV Irradiation

Normal Adult Human Dermal Fibroblasts (NHDF-Ad) were seeded in 96-well plates at the density of 5 × 10^4^ cells/well and incubated in standard DMEM medium supplemented with FBS and antibiotic solution. The plates were incubated at 37 °C in 5% CO_2_ for 24 h to allow attachment to the bottom of the wells. After 24 h, the medium was changed to medium supplemented with folic acid at the concentrations: 0 μM (control) and 0.01%. After 24 h incubation with 0.01% folic acid, the cells were treated with UV irradiation (Balance Facial Solarium GB 2000, Luxoplast Kunststofftechnik GmbH; Ampfing, Germany) for 15 min. After incubation for 24 h, 48 h, or 72 h, WST-1 tests were conducted according to the manufacturer’s protocol.

### 4.5. Influence of UV Radiation on the mRNA Expression of Selected Genes That Regulate the Cell Cycle CCND1, P53, BAX, and BCL-2 in Cells Protected by Folic Acid

To evaluate the expression of the CCND1, P53, BAX, and BCL-2 genes, cells were seeded at a density of 5 × 10^5^ onto 21.5 cm2 (60 mm diameter) cell culture dishes (Nunc International, Rochester, NY, USA) and grown for 48 h. After 24 h of incubation in standard culture conditions (37 °C, 5% CO_2_, and 95% humidity), the medium DMEM with 10% FBS and 1% pen/strep was changed to medium supplemented with 0.01% folic acid. At 24 h after medium change, NHDF cells were irradiated 15 min by UV light. RNA extraction was conducted the day after cells UV irradiation. Control group of cells in this study constituted: NHDF cells cultured in DMEM, supplemented with 1% penicillin/streptomycin solution, in atmosphere of 95% humidity and 5% CO_2_ not treated with UV, NHDF cells under standard culture condition not treated with FA, irradiated 15 min by UV, and NHDF cell culture supplemented with 0.01% FA.

Detection of the mRNA expression of the examined genes was carried out using a RT-qPCR technique with SYBR Green chemistry (SensiFastTM SYBR Green No-ROX One-Step) (Bioline, Meridian Bioscience, Cincinnati, OH, USA) and CFX Connect Real-Time PCR Detection System (Bio-Rad, Hercules, CA, USA). Total RNA was extracted from cells with the use of Quick-RNA™ MiniPrep (cat. No. R1055, Zymo Research Corp., Irvine, CA, USA) according to the manufacturer’s protocol.

RNA concentration and purity ratio (260/280) were measured spectrophotometrically (Shimadzu UV-1800 spectrophotometer, Kyoto, Japan). The primers for CCND1, P53, BAX, and BCL-2 mRNAs were synthesized in Oligo.pl at the Institute of Biochemistry and Biophysics of the Polish Academy of Sciences (Warsaw, Poland). The sequences of the primers used in RT-qPCR are as follows (PF—Primer Forward sequence; PR—Primer Reverse sequence): P53 (PF: 5′-TAACAgTTCCTgCATgggCggC-3′; PR: 5′-AggACAggCACAAACACgCACC-3′), BAX (PF: 5′-CCTgTgCACCAaggTgCCggAACT-3′; PR: 5′-CCACCCTggTCTTggATCCAgCCC-3′), BCL-2 (PF: 5′-TTgTggCCTTCTTTgAgTTCggTg-3′; PR: 5′–ggTgCCggTT CAggTACTCAgTCA-3′), CCND1 (PF: 5′-gAgCTgCTCCTggTgAACAAg-3′; PR: 5′-gTgTTTgCggATgATCTgTTTg-3′), and β-actin (PF: 5′-TCACCCACACTgTgCCCATCTACgA-3′; PR: 5′-CAgCggAACCgCTCATTgCCAATgg-3′).

The thermal profile for RT-qPCR was as follows: 45 °C for 10 min for reverse transcription and 95 °C for 2 min, followed by 45 cycles at 95 °C for 5 s, 60 °C for 10 s, and 72 °C for 5 s for amplification. Each gene expression analysis was performed in triplicate. A melting-curve analysis was performed to confirm the RT-qPCR specificity. Following RT-PCR, the samples were subjected to temperature ramp from 60 °C to 95 °C at the rate of 0.2 °C/s, with continuous fluorescence monitoring for melting curve analysis. The results were analyzed using a Bio-Rad CFX Manager v.3.1 provided by BIO-RAD Laboratories, (Hercules, California, USA).

The relative gene expression was obtained after normalization with the endogenous human β-actin, and the difference in threshold cycle (Ct) between the treated and untreated cells was determined using the 2^−ΔΔCt^ formula.

### 4.6. Measurement of the Effect of UV Radiation on the Interaction Between Folic Acid and Free Radicals Using Electron Paramagnetic Resonance (EPR)

Interactions with free radicals of nonirradiated and UV-irradiated samples of folic acid were tested. The time of irradiation was 15 min. The irradiation was performed by lamp emitting UVB and UVA light (Balance Facial Solarium GB 2000, Luxoplast Kunststofftechnik GmbH; Ampfing, Germany). The UVB/UVA radiation was performed from the distance of lamp to the sample of 30 cm.

For the EPR measurements, 10% ethyl alcohol solutions of DPPH (2,2-diphenyl-1-picrylhydrazyl radical), the reference probe of free radicals, were prepared. The nonirradiated folic acids with concentrations of 0.1%; 0.01%; 0.001%; 0.0001%; and 0.00001% to the 10% ethanol solutions of DPPH were added. Next, the same solutions were prepared with UV-irradiated folic acids. EPR spectra were measured for the samples placed in the thin-walled glass tubes with external diameter of 1 mm. DPPH was used as the reference to examine the interactions of folic acid with free radicals. These interactions of the tested folic acid samples caused the decrease in EPR line of DPPH.

Electron paramagnetic resonance measurements were performed using an X band EPR spectrometer, with microwave frequency of 9.3 GHz produced by Radiopan Firm (Poznań, Poland). A magnetic modulation of 100 kHz was used. Microwave frequency was measured by MCM101 recorder of Eprad Firm (Poznań, Poland). The modulation of magnetic field was 100 kHz. The EPR spectra were measured as first-derivative lines by the use of Rapid Scan Unit of Jagmar Firm (Kraków, Poland), which was used together with the EPR spectrometer. The individual EPR spectrum was numerically collected during time of 1 s. All the EPR spectra were measured with low microwave power of 2.2 mW to avoid microwave saturation effect of resonance lines. The total microwave power produced by klystron of the spectrometer was 70 mW. The application of 15 dB attenuation made it possible to obtain the low microwave power to 2.2 mW.

The numerical acquisition of EPR lines and the spectral analyses were performed by the use of programs of Jagmar Firm (Kraków, Poland) and LabVIEW 8.5.1 (National Instruments Corporation, Austin, TX, USA).

**Figure 6:**
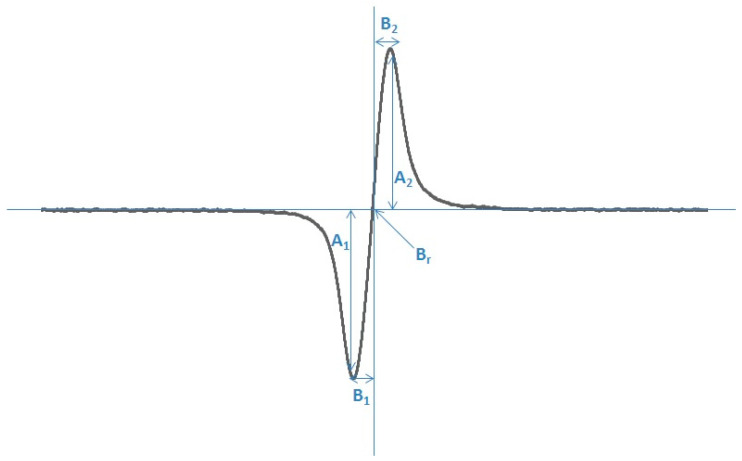
EPR spectrum of DPPH in ethanol solution with folic acid. The parameters of the spectrum: amplitude (A), linewidth (ΔB_pp_), B_r_—magnetic induction, A_1_/A_2_ and B_1_/B_2_ asymmetry parameters.

The following parameters of EPR spectra were analyzed: amplitudes (A), and linewidths (ΔB_pp_), g-factors, A_1_/A_2_, and B_1_/B_2_. These parameters are shown in Figure 6. Amplitude (A) is proportional to the free radical concentration in the samples. Linewidth (ΔB_pp_) depends on magnetic interactions in the samples. Dipolar interactions of free radicals increase the linewidth [107,108].

g-Factors were calculated from the paramagnetic resonance condition according to the formula [13]: g = hν /μ_B_B_r_, where h—Planck constant, ν—microwave frequency, μ_B_—Bohr magneton, and B_r_—induction of resonance magnetic field.

A_1_/A_2_ and B_1_/B_2_ are line-shape parameters, which show the asymmetry of the EPR lines. For symmetric spectra, both parameters A_1_/A_2_ and B_1_/B_2_ reveal a value of 1. Values different from 1 indicate asymmetry of the spectra.

### 4.7. Statistical Analysis

A one-way ANOVA followed by Tukey’s test was used to determine significant differences between the mean of viability control cells (cells not treated with folic acid) and folic acid treated cells. The data are presented as the means ± SD. The fold change (2^−ΔΔCt^) method was used to present the RT-qPCR results. Values of *p* < 0.05 were considered statistically significant. Statistical analysis was carried out using TIBCO Statistica 13.6.0 software.

## 5. Conclusions

Molecular and EPR studies both confirm the effectiveness of folic acid as a protective ingredient in dermo cosmetics and pharmaceutical products. However, given the susceptibility of folic acid to photodegradation and the negative effects of its decomposition products, it is necessary to consider using other UV protectors alongside it. Further research in this area may yield valuable solutions for pharmacotherapy and cosmetology.

## Figures and Tables

**Table 1:** The parameters amplitudes (A), linewidths (ΔB_pp_), g-factors, A_1_/A_2_, and B_1_/B_2_ of the analyzed EPR spectra of DPPH in ethyl alcohol solution with nonirradiated folic acid. The parameters are defined in Figure 1. The concentrations of folic acid in ethyl alcohol were written in 10%. FA: folic acid, DPPH: 2,2-diphenyl-1-picrylhydrazyl radical.

Sample	A [a.u.] [+0.01]	ΔB_pp_ [mT] [+0.02]	g [+0.0002]	A_1_/A_2_ [+0.02]	B_1_/B_2_ [+0.02]
DPPH	0.40	0.48	2.0036	1.00	1.25
0.1% FA	0.27	0.48	2.0036	0.93	1.15
0.01%	0.24	0.47	2.0036	0.72	1.32
0.001%	0.27	0.47	2.0036	0.98	1.25
0.0001%	0.27	0.47	2.0036	0.98	1.25
0.00001%	0.33	0.45	2.0036	0.99	1.17

**Table 2:** The parameters amplitudes (A), linewidths (ΔB_pp_), g-factors, A_1_/A_2_, and B_1_/B_2_ of the analyzed EPR spectra of DPPH in ethyl alcohol solution with UV-irradiated folic acid. The parameters are defined in Figure 1. The concentrations of folic acid in ethyl alcohol were written in 10%.

Sample	A [a.u.] [+0.01]	ΔB_pp_ [mT] [+0.02]	g [+0.0002]	A_1_/A_2_ [+0.02]	B_1_/B_2_ [+0.02]
DPPH	0.40	0.48	2.0036	1.00	1.25
0.1%	0.77	0.47	2.0036	0.90	1.02
0.01%	0.41	0.48	2.0036	0.98	1.15
0.001%	0.45	0.46	2.0036	0.87	1.06
0.0001%	0.50	0.47	2.0036	0.92	1.34
0.00001%	0.47	0.47	2.0036	0.89	1.37

## Data Availability

Data are contained within the article.

## References

[1] Balashova O.A., Visina O., Borodinsky L.N. (2018). Folate action in nervous system development and disease. Dev. Neurobiol..

[2] Cieślik E., Cieślik I. (2018). Occurrence and Significance of Folic Acid. Pteridines.

[3] Kurowska K., Kobylinska M., Antosik K. (2023). Folic acid-Importane for human health and its role in COVID-19 therapy. Rocz. Państwowego Zakładu Hig..

[4] Witthöft C.M., Forssén K., Johannesson L., Jägerstad M. (1999). Folates—Food sources, analyses, retention and bioavailability. Food Nutr. Res..

[5] Strickland K.C., Krupenko N.I., Krupenko S.A. (2013). Molecular mechanisms underlying the potentially adverse effects of folate. Clin. Chem. Lab. Med..

[6] Stanger O. (2002). Physiology of folic acid in health and disease. Curr. Drug Metab..

[7] Crider K.S., Yang T.P., Berry R.J., Bailey L.B. (2012). Folate and DNA methylation: A review of molecular mechanisms and the evidence for folate’s role. Adv. Nutr..

[8] Safi J., Joyeux L., Chalouhi G.E. (2012). Periconceptional folate deficiency and implications in neural tube defects. J. Pregnancy.

[9] Fardous A.M., Heydari A.R. (2023). Uncovering the Hidden Dangers and Molecular Mechanisms of Excess Folate: A Narrative Review. Nutrients.

[10] Dębowska R., Rogiewicz K., Iwanenko T., Kruszewski M., Eris I. (2005). Folic Acid (Folacin)–New application of a cosmetic ingredient. Kosmet. Med..

[11] Eris I., Dębowska R. (2003). Kwas foliowy (folacyna) w preparatach do pielęgnacji twarzy—Ocena działania witaminy na komórki skóry w badaniach in vitro. Dermatologica.

[12] Rabe J.H., Mamelak A.J., McElgunn P.J., Morison W.L., Sauder D.N. (2006). Photoaging: Mechanisms and repair. J. Am. Acad. Dermatol..

[13] Bickers D.R., Athar M. (2006). Oxidative Stress in the Pathogenesis of Skin Disease. J. Investig. Dermatol..

[14] Yaar M., Gilchrest B.A. (2007). Photoageing: Mechanism, prevention and therapy. Br. J. Dermatol..

[15] Ittycheri A., Lipsky Z.W., Hookway T.A., German G.K. (2023). Ultraviolet light induces mechanical and structural changes in full thickness human skin. J. Mech. Behav. Biomed. Mater..

[16] Svobodova A., Walterova D., Vostalova J. (2006). Ultraviolet Light Induced Alteration to the Skin. Biomed. Pap. Med. Fac. Univ. Palacky Olomouc Czech. Repub..

[17] Palmer D.M., Kitchin J.S. (2010). Oxidative damage, skin aging, antioxidants and a novel antioxidant rating system. J. Drugs Dermatol..

[18] Pinnell S.R. (2003). Cutaneous photodamage, oxidative stress, and topical antioxidant protection. J. Am. Acad. Dermatol..

[19] Borradale D.C., Kimlin M.G. (2012). Folate degradation due to ultraviolet radiation: Possible implications for human health and nutrition. Nutr. Rev..

[20] Hirakawa K., Suzuki H., Oikawa S., Kawanishi S. (2003). Sequence-specific DNA damage induced by ultraviolet A-irradiated folic acid via its photolysis product. Arch. Biochem. Biophys..

[21] Akhtar M.J., Khan M.A., Ahmad I. (2003). Identification of photoproducts of folic acid and its degradation pathways in aqueous solution. J. Pharm. Biomed. Anal..

[22] Thomas A.H., Suarez G., Cabrerizo F.M., Martino R., Capparelli A.L. (2000). Study of the photolysis of folic acid and 6-formylpterin in acid aqueous solutions. J. Photochem. Photobiol..

[23] Off M.K., Steindal A.E., Porojnicu A.C., Juzeniene A., Vorobey A., Johnsson A., Moan J. (2005). Ultraviolet photodegradation of folic acid. J. Photochem. Photobiol. B.

[24] Goossens J.F., Thuru X., Bailly C. (2021). Properties and reactivity of the folic acid and folate photoproduct 6-formylpterin. Free Radic. Biol. Med..

[25] Carvalho C., Silva R., Melo T.M.V.D.P.e., Inga A., Saraiva L. (2024). P53 and the Ultraviolet Radiation-Induced Skin Response: Finding the Light in the Darkness of Triggered Carcinogenesis. Cancers.

[26] Wiman K.G., Zhivotovsky B. (2017). Understanding cell cycle and cell death regulation provides novel weapons against human diseases. J. Intern. Med..

[27] Benjamin C.L., Ullrich S.E., Kripke M.L., Ananthaswamy H.N. (2008). P53 Tumor Suppressor Gene: A Critical Molecular Target for UV Induction and Prevention of Skin Cancer. Photochem. Photobiol..

[28] Feroz W., Sheikh A.M.A. (2020). Exploring the multiple roles of guardian of the genome: P53. Egypt. J. Med. Hum. Genet..

[29] Kulsoom B., Shamsi T.S., Afsar N.A., Memon Z., Ahmed N., Hasnain S.N. (2018). Bax, Bcl-2, and Bax/Bcl-2 as prognostic markers in acute myeloid leukemia: Are we ready for Bcl-2-directed therapy?. Cancer Manag. Res..

[30] Georgakopoulou E., Evangelou K., Havaki S., Townsend P., Kanavaros P., Gorgoulis V.G. (2016). Apoptosis or senescence? Which exit route do epithelial cells and fibroblasts preferentially follow? Mech. Ageing Dev..

[31] Solaki M., Ewald J.C. (2018). Fueling the Cycle: CDKs in Carbon and Energy Metabolism. Front. Cell Dev. Biol..

[32] Fajas L. (2013). Re-thinking cell cycle regulators: The cross-talk with metabolism. Front. Oncol..

[33] Alao J.P., Gamble S.C., Stavropoulou A.V., Pomeranz K.M., Lam E., Coombes R.C., Vigushin D.M. (2006). The cyclin D1 proto-oncogene is sequestered in the cytoplasm of mammalian cancer cell lines. Mol. Cancer.

[34] Hao Q., Chen J., Lu H., Zhou X., Yao X. (2022). The ARTS of P53-dependent mitochondrial apoptosis. J. Mol. Cell Biol..

[35] Yang K., Hitomi M., Stacey D.W. (2006). Variations in cyclin D1 levels through the cell cycle determine the proliferative fate of a cell. Cell Div..

[36] Smith J., Rai V. (2024). Novel Factors Regulating Proliferation, Migration, and Differentiation of Fibroblasts, Keratinocytes, and Vascular Smooth Muscle Cells during Wound Healing. Biomedicines.

[37] Pondeljak N., Lugović-Mihić L., Tomić L., Parać E., Pedić L., Lazić-Mosler E. (2023). Key Factors in the Complex and Coordinated Network of Skin Keratinization: Their Significance and Involvement in Common Skin Conditions. Int. J. Mol. Sci..

[38] Moltrasio C., Romagnuolo M., Marzano A.V. (2022). Epigenetic Mechanisms of Epidermal Differentiation. Int. J. Mol. Sci..

[39] Eckert R.L., Efimova T., Dashti S.R., Balasubramanian S., Deucher A., Crish J.F., Sturniolo M., Bone F. (2002). Keratinocyte survival, differentiation, and death: Many roads lead to mitogen-activated protein kinase. J. Investig. Dermatol. Symp. Proc..

[40] Eckhart L., Declercq W., Ban J., Rendl M., Lengauer B., Mayer C., Lippens S., Vandenabeele P., Tschachler E. (2000). Terminal differentiation of human keratinocytes and stratum corneum formation is associated with caspase-14 activation. J. Investig. Dermatol..

[41] Eckhart L., Lippens S., Tschachler E., Declercq W. (2013). Cell death by cornification. Biochim. Biophys. Acta..

[42] Bito T., Nishigori C. (2012). Impact of reactive oxygen species on keratinocyte signaling pathways. J. Dermatol. Sci..

[43] Rinnerthaler M., Bischof J., Streubel M.K., Trost A., Richter K. (2015). Oxidative stress in aging human skin. Biomolecules.

[44] D’Errico M., Lemma T., Calcagnile A., Proietti De Santis L., Dogliotti E. (2007). Cell type and DNA damage specific response of human skin cells to environmental agents. Mutat. Res..

[45] Badr-Eldin S.M., Aldawsari H.M., Kotta S., Deb P.K., Venugopala K.N. (2022). Three-Dimensional In Vitro Cell Culture Models for Efficient Drug Discovery: Progress So Far and Future Prospects. Pharmaceuticals.

[46] Bissell M.J., Rizki A., Mian I.S. (2003). Tissue architecture: The ultimate regulator of breast epithelial function. Curr. Opin. Cell Biol..

[47] Riabinin A., Pankratova M., Rogovaya O., Vorotelyak E., Terskikh V., Vasiliev A. (2024). Ideal Living Skin Equivalents, from Old Technologies and Models to Advanced Ones: The Prospects for an Integrated Approach. BioMed Res. Int..

[48] https://www.ncbi.nlm.nih.gov/books/NBK304366/.

[49] Tang X., Yang T., Yu D., Xiong H., Zhang S. (2024). Current insights and future perspectives of ultraviolet radiation (UV) exposure: Friends and foes to the skin and beyond the skin. Environ. Int..

[50] Battie C., Verschoore M. (2012). Cutaneous solar ultraviolet exposure and clinical aspects of photodamage. Indian J. Dermatol. Venereol. Leprol..

[51] Davis A.E., Kennelley G.E., Amaye-Obu T., Jowdy P.F., Ghadersohi S., Nasir-Moin M., Paragh G., Berman H.A., Huss W.J. (2024). The phenomenon of phototoxicity and long-term risks of commonly prescribed and structurally diverse drugs. J. Photochem. Photobiol..

[52] Basu-Modak S., Tyrrell R.M., Giacomoni P.U. (2001). Modulation of gene expression by solar ultraviolet radiation. Comprehensive Series in Photosciences.

[53] Li Q., Qian W., Zhang Y., Hu L., Chen S., Xia Y. (2023). A new wave of innovations within the DNA damage response. Signal Transduct. Target. Ther..

[54] van Heemst D., den Reijer P.M., Westendorp R.G. (2007). Ageing or cancer: A review on the role of caretakers and gatekeepers. Eur. J. Cancer..

[55] Kciuk M., Marciniak B., Mojzych M., Kontek R. (2020). Focus on UV-Induced DNA Damage and Repair—Disease Relevance and Protective Strategies. Int. J. Mol. Sci..

[56] Averill-Bates D. (2024). Reactive oxygen species and cell signaling. Review. Biochim. Biophys. Acta Mol. Cell Res..

[57] https://dermnetnz.org/cme/phototherapy/uva-photochemotherapy.

[58] Young A.R., Giacomoni P.U., Jori G., Hader D. (2007). Biophysical and Physiological Effects of Solar Radiation on Human Skin.

[59] Godar D.E. (2005). UV doses worldwide. Photochem. Photobiol..

[60] https://ec.europa.eu/health/scientific_committees/scheer/docs/scheer_o_003.pdf.

[61] https://www.ncbi.nlm.nih.gov/books/NBK321117/.

[62] Tanveer M.A., Rashid H., Tasduq S.A. (2023). Molecular basis of skin photoaging and therapeutic interventions by plant-derived natural product ingredients: A comprehensive review. Heliyon.

[63] Young O., Ngo N., Lin L., Stanbery L., Creeden J.F., Hamouda D., Nemunaitis J. (2023). Folate Receptor as a Biomarker and Therapeutic Target in Solid Tumors. Curr. Probl. Cancer..

[64] Elnakat H., Ratnam M. (2004). Distribution, functionality and gene regulation of folate receptor isoforms: Implications in targeted therapy. Adv. Drug Deliv. Rev..

[65] Salazar M.D., Ratnam M. (2007). The folate receptor: What does it promise in tissue-targeted therapeutics?. Cancer Metastasis Rev..

[66] Scaranti M., Cojocaru E., Banerjee S., Banerji U. (2020). Exploiting the folate receptor α in oncology. Nat. Rev. Clin. Oncol..

[67] Yi Y.S. (2016). Folate Receptor-Targeted Diagnostics and Therapeutics for Inflammatory Diseases. Immune Netw..

[68] Hsueh M.F., Lu Y., Wheeler L., Wellman S.S., Bolognesi M.P., Kraus V.B. (2017). Functional folate receptor cells within synovium and fluid as therapeutic targets for osteoarthritis. Osteoarthr. Cartil..

[69] Halik P.K., Koźmiński P., Gniazdowska E. (2021). Perspectives of Methotrexate-Based Radioagents for Application in Nuclear Medicine. Mol. Pharm..

[70] Lee W.D., Pirona A.C., Sarvin B., Stern A., Nevo-Dinur K., Besser E., Sarvin N., Lagziel S., Mukha D., Raz S. (2021). Tumor Reliance on Cytosolic versus Mitochondrial One-Carbon Flux Depends on Folate Availability. Cell Metab..

[71] Valerio H.P., Ravagnani F.G., Ronsein G.E., Di Mascio P. (2021). A Single Dose of Ultraviolet-A Induces Proteome Remodeling and Senescence in Primary Human Keratinocytes. Sci. Rep..

[72] Yang T.T., Lan C.E. (2025). Photocarcinogenesis of the skin: Current status and future trends. Kaohsiung J. Med. Sci..

[73] Rastogi R.P., Richa Kumar A., Tyagi M.B., Sinha R.P. (2010). Molecular mechanisms of ultraviolet radiation-induced DNA damage and repair. J. Nucl. Acids..

[74] Cadet J., Grand A., Douki T. (2015). Solar UV radiation-induced DNA bipyrimidine photoproducts: Formation and mechanistic insights. Top. Curr. Chem..

[75] Wong H.Y., Lee R., Chong S., Kapadia S., Freeman M., Murigneux V., Brown S., Soyer H.P., Roy E., Khosrotehrani K. (2023). Epidermal mutation accumulation in photodamaged skin is associated with skin cancer burden and can be targeted through ablative therapy. Sci. Adv..

[76] Rosette C., Karin M. (1996). Ultraviolet Light and Osmotic Stress: Activation of the JNK Cascade Through Multiple Growth Factor and Cytokine Receptors. Science.

[77] Rehemtulla A., Hamilton C.A., Chinnaiyan A.M., Dixit V.M. (1997). Ultraviolet radiation-induced apoptosis is mediated by activation of CD-95 (Fas/APO-1). J. Biol. Chem..

[78] Batista L.F.Z., Kaina B., Meneghini R., Menck C.F.M. (2009). How DNA lesions are turned into powerful killing structures: Insights from UV-induced apoptosis. Mutat. Res..

[79] Lee C.H., Wu S.B., Hong C.H., Yu H.S., Wei Y.H. (2013). Molecular Mechanisms of UV-Induced Apoptosis and Its Effects on Skin Residential Cells: The Implication in UV-Based Phototherapy. Int. J. Mol. Sci..

[80] Wang Y., Rosenstein B., Goldwyn S., Zhang X., Lebwohl M., Wei H. (1998). Differential regulation of P53 and Bcl-2 expression by ultraviolet A and B. J. Investig. Dermatol..

[81] Susnow N., Zeng L., Margineantu D., Hockenbery D.M. (2009). Bcl-2 family proteins as regulators of oxidative stress. Semin. Cancer Biol..

[82] Longoni B., Boschi E., Demontis G.C., Marchiafava P.L., Mosca F. (1999). Regulation of Bcl-2 protein expression during oxidative stress in neuronal and in endothelial cells. Biochem. Biophys. Res. Commun..

[83] Deng G., Su J.H., Ivins K.J., Van Houten B., Cotman C.W. (1999). Bcl-2 facilitates recovery from DNA damage after oxidative stress. Exp. Neurol..

[84] Kowaltowski A.J., Fenton R.G., Fiskum G. (2004). Bcl-2 family proteins regulate mitochondrial reactive oxygen production and protect against oxidative stress. Free Radic. Biol. Med..

[85] Rieger K.E., Chu G. (2004). Portrait of transcriptional responses to ultraviolet and ionizing radiation in human cells. Nucleic Acids Res..

[86] Alamro A.A., Al-Malky M.M., Ansari M.G.A., Amer O.E., Alnaami A.M., Hussain S.D., Barhoumi T.A., Alghamdi A.A., Haq S.H., Sabico S. (2021). The effects of melatonin and vitamin D3 on the gene expression of Bcl-2 and BAX in MCF-7 breast cancer cell line. J. King Saud. Univ. Sci..

[87] Montalto F.I., De Amicis F. (2020). Cyclin D1 in Cancer: A Molecular Connection for Cell Cycle Control, Adhesion and Invasion in Tumor and Stroma. Cells.

[88] Balagula Y., Kang S., Patel M.J. (2015). Synergism between mTOR pathway and ultraviolet radiation in the pathogenesis of squamous cell carcinoma and its implication for solid-organ transplant recipients. Photodermatol. Photoimmunol. Photomed..

[89] https://www.cdc.gov/radiation-health/features/uv-radiation.html.

[90] https://ec.europa.eu/health/ph_risk/committees/04_sccp/docs/sccp_oc03_019.pdf.

[91] Ishii H., Arai T., Mori H., Yamada H., Endo N., Makino K., Fukuda K. (2005). Protective effects of intracellular reactive oxygen species generated by 6-formylpterin on tumor necrosis factor-alpha-induced apoptotic cell injury in cultured rat hepatocytes. Life Sci..

[92] Funakoshi T., Miyata H., Imoto T., Arai T., Endo N., Makino K., Yang C.H., Ohama E. (2003). 6-Formylpterin protects retinal neurons from transient ischemia-reperfusion injury in rats: A morphological and immunohistochemical study. Neuropathology.

[93] Kim H.J., Jin S.P., Kang J., Bae S.H., Son J.B., Oh J.H., Youn H., Kim S.K., Kang K.W., Chung J.H. (2024). Uncovering the impact of UV radiation on mitochondria in dermal cells: A STED nanoscopy study. Sci. Rep..

[94] Sabetghadam Moghadam M., Wiens E., Gauvrit S., Sammynaiken R., Collins M.M. (2025). Electron paramagnetic resonance spectroscopy for analysis of free radicals in zebrafish. PLoS ONE.

[95] Nakanishi I., Shoji Y., Ohkubo K., Ito H., Fukuzumi S. (2023). Water-Induced Regeneration of a 2,2-Diphenyl-1-picrylhydrazyl Radical after Its Scandium Ion-Promoted Electron-Transfer Disproportionation in an Aprotic Medium. Molecules.

[96] Akhtar M.J., Khan M.A., Ahmad I. (1999). Photodegradation of folic acid in aqueous solution. J. Pharm. Biomed. Anal..

[97] Fukuwatari T., Fujita M., Shibata K. (2009). Effects of UVA irradiation on the concentration of folate in human blood. Biosci. Biotechnol. Biochem..

[98] Rashed A. (2024). High-Performance Liquid Chromatography (HPLC): Principles, Applications, Versatality, Efficiency, Innovation and Comparative Analysis in Modern Analytical Chemistry and In Pharmaceutical Sciences. Clin. Investig..

[99] Boulgakov A.A., Moor S.R., Jo H.H., Metola P., Joyce L.A., Marcotte E.M., Welch C.J., Anslyn E.V. (2020). Next-Generation TLC: A Quantitative Platform for Parallel Spotting and Imaging. J. Org. Chem..

[100] Lefeuvre S., Bois-Maublanc J., Mongeois E., Policarpo V., Formaux L., Francia T., Billaud E.M., Got L. (2021). Quantitation using HRMS: A new tool for rapid, specific and sensitive determination of catecholamines and deconjugated methanephrines metanephrines in urine. J. Chromatogr. B Analyt Technol. Biomed. Life Sci..

[101] Juzeniene A., Thu Tam T.T., Iani V., Moan J. (2013). The action spectrum for folic acid photodegradation in aqueous solutions. J. Photochem. Photobiol. B..

[102] Joshi R., Adhikari S., Patro B.S., Chattopadhyay S., Mukherjee T. (2001). Free radical scavenging behavior of folic acid: Evidence for possible antioxidant activity. Free Radic. Biol. Med..

[103] Gliszczyńska-Świgło A. (2007). Folates as antioxidants. Food Chem..

[104] Tejchman K., Kotfis K., Sieńko J. (2021). Biomarkers and Mechanisms of Oxidative Stress—Last 20 Years of Research with an Emphasis on Kidney Damage and Renal Transplantation. Int. J. Mol. Sci..

[105] Asbaghi O., Ghanavati M., Ashtary-Larky D., Bagheri R., Rezaei Kelishadi M., Nazarian B., Nordvall M., Wong A., Dutheil F., Suzuki K. (2021). Effects of Folic Acid Supplementation on Oxidative Stress Markers: A Systematic Review and Meta-Analysis of Randomized Controlled Trials. Antioxidants.

[106] Liguori I., Russo G., Curcio F., Bulli G., Aran L., Della-Morte D., Gargiulo G., Testa G., Cacciatore F., Bonaduce D. (2018). Oxidative stress, aging, and diseases. Clin. Interv. Aging.

[107] Eaton G.R., Eaton S.S., Salikhov K.M. (1998). Foundations of Modern EPR.

[108] Wertz J.E., Bolton J.R. (1986). Electron Spin Resonance: Elementary Theory and Practical Applications.

